# Supplementary material for: Human Dendritic Cells Transmit Enterovirus A71 via Heparan Sulfates to Target Cells Independent of Viral Replication
Source: Microbiol Spectr. 2022 Oct 12;10(6):e02822-22. doi: 10.1128/spectrum.02822-22 (PMC9769767; doi:10.1128/spectrum.02822-22)
Supplement: Supplemental file 1 — Fig. S1 and S2. Download spectrum.02822-22-s0001.pdf, PDF file, 0.7 MB [file spectrum.02822-22-s0001.pdf]

Supplementary figures

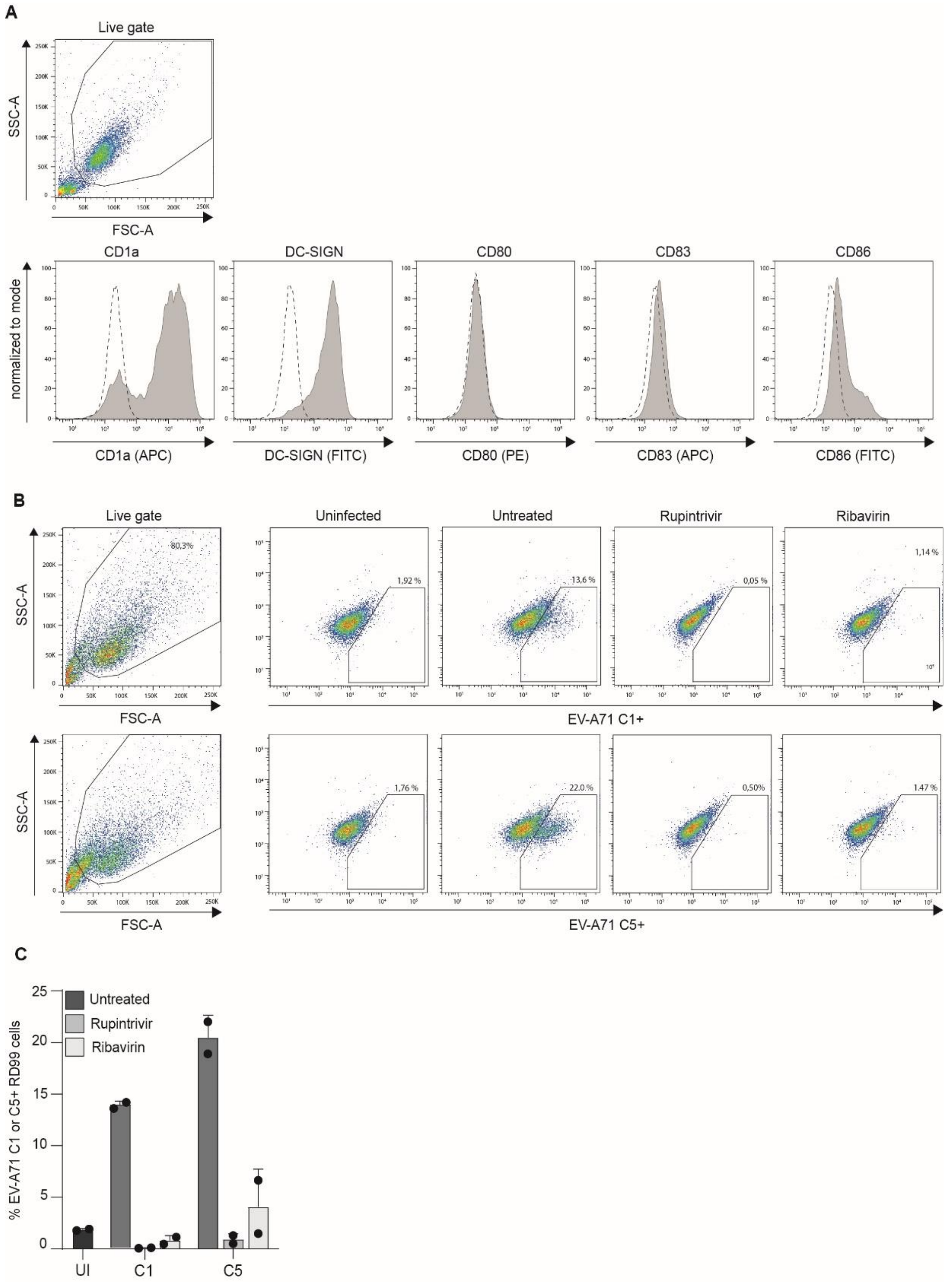

Supplementary figure 1 | A. Expression of DC-markers CD1a and DC-SIGN, and maturation markers CD80, CD83, and CD86 was determined by flow cytometry. (A) Populations plots and histograms of surface makers for one representative DC donor are shown to indicate the characterization of DCs. (B-C) RD99 cells were exposed to EV-A71 C1 or C5 in the presence of rupintrivir (1 µM) or ribavirin (10 µM). The percentage of EV-A71 positive RD99 cells was determined by flow cytometry.

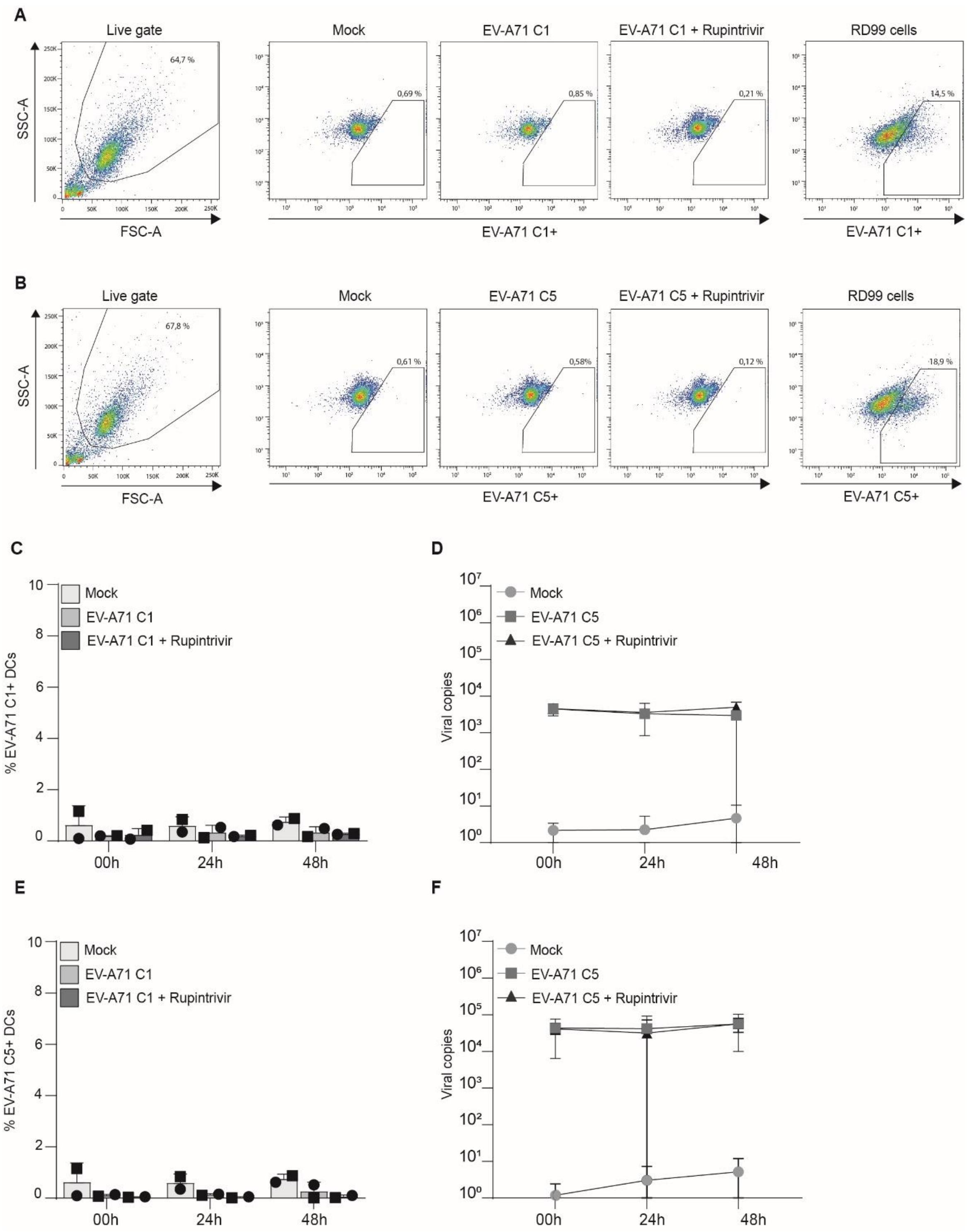

Supplementary figure 2 | A-F. DCs were exposed to EV-A71 C1 or C5 (MOI 0.5) for two days in the presence or absence of rupintrivir (1 µM). Cells and supernatant were collected at different time points and analysed by flow cytometry and RT-qPCR, respectively. Direct infection (24h) of RD99 cells was taken along as a positive control. Flow cytometry analyses of DC infection is shown for one representative donor (A and B) and combined data for different donors on infection as determined by flow cytometry (C,E, N=2) or quantitative PCR (D,F, N=2). The symbols represent independent donors mean ± s.d. (flow cytometry) or mean ± SEM (RT-qPCR) of duplicates.
